# Health-related quality of life and mental well-being of healthy and diseased persons in 8 countries: Does stringency of government response against early COVID-19 matter?

**DOI:** 10.1016/j.ssmph.2021.100913

**Published:** 2021-09-01

**Authors:** Di Long, Juanita A. Haagsma, Mathieu F. Janssen, John N. Yfantopoulos, Erica I. Lubetkin, Gouke J. Bonsel

**Affiliations:** a Department of Public Health, Erasmus MC, Rotterdam, the Netherlands; b Section Medical Psychology and Psychotherapy, Department of Psychiatry, Erasmus MC, Rotterdam, the Netherlands; c MBA - Health Department of Economics, National and Kapodistrian University of Athens, Athens, Greece; d Department of Community Health and Social Medicine, CUNY School of Medicine, New York, NY, USA; e EuroQol Research Foundation, Rotterdam, the Netherlands

**Keywords:** HRQoL, Well-being, EQ-5D-5L, WHO-5, COVID-19, Stringency index

## Abstract

**Objectives:**

Our study aimed to (1) assess health-related quality of life (HRQoL) and mental well-being of healthy and diseased persons in the general population during the early stage of the COVID-19 pandemic and (2) examine the relationship between HRQoL and mental well-being and individual characteristics and government response against COVID-19, as measured by the stringency index.

**Methods:**

A web-based survey was administered to a cohort of persons from the general population of eight countries: Greece, Italy, the Netherlands, Russia, South Africa, Sweden, the United Kingdom (UK) and the United States of America (US) from April 22 to May 5 and May 26 to June 1, 2020. Country-level stringency indices were adopted from the COVID-19 Government Response Tracker. Primary outcomes were HRQoL, measured using the EQ-5D-5L, and mental well-being, measured using the World Health Organization-5 Well-Being (WHO-5).

**Findings:**

21,354 respondents were included in the study. Diseased respondents had lower EQ-5D-5L and WHO-5 scores compared to healthy respondents. Younger respondents had lower WHO-5 scores than older respondents. The stringency index had a stronger association with the EQ-5D-5L and WHO-5 among diseased respondents compared to healthy respondents. Increasing stringency was associated with an increase in EQ-5D-5L scores but a decrease in the WHO-5 index.

**Conclusion:**

The stringency of government response is inversely related to HRQoL and mental well-being with a small positive relation with HRQoL and strong negative relation with mental well-being. The magnitude of effects differed for healthy and diseased persons and by age but was most favourable for diseased and older persons.

## Highlights

• Persons with chronic conditions scored lower on EQ-5D-5L and WHO-5 domains, resulting in worse HRQoL and mental well-being.
• An age gradient was found where younger persons had worse mental well-being than older persons.
• Stringency of government response had opposite patterns for HRQoL and mental well-being.
• The magnitude of effects of stringency differed for healthy and diseased persons but was most favourable for diseased persons.
• Not feeling protected against COVID-19 had the one of the most negative association with mental well-being

## 1 Introduction

COVID-19 is an infectious disease that, after being initially identified in China in December 2019, has resulted in an ongoing pandemic. An array of direct and indirect health impacts has emerged from the COVID-19 pandemic. Direct impact results from the infection itself (Chenet al., 2020), whereas indirect effects may have multiple sources, such as fear of infection (Lazzerini et al., 2020), stigma (Ahorsu et al., 2020; Logie & Turan, 2020), and anxiety or stress (Rossiet al., 2020; Salariet al., 2020). Furthermore, the psychological impact of a given government's response against COVID-19, such as quarantine, social distancing, and lockdown restrictions may affect health, too. While protective for the spread of the infection, these measures may yield ill-health effects (Serafini et al., 2020). The lack of clarity concerning when these measures will expire further adds to this uncertainty (Bakioglu et al., 2020). Moreover, the COVID-19 pandemic has severely disrupted education and the economy, resulting in the loss of jobs and productivity (Crayne, 2020), usual health care (Woolhandler & Himmelstein, 2020; Yadav et al., 2020), and a greater number of persons living in poverty (Sumner et al., 2020). The disruption results in a further increase in health inequalities across socioeconomic groups around the world (Ahmed et al., 2020).

To date, investigators have revealed that the indirect impact of COVID-19 differs for subgroups within the general population. For example, people with pre-existing physical and psychiatric chronic comorbidities have been found to be more susceptible to COVID-19 infection (Banerjeeet al., 2020; Zhouet al.eng, 2020), as well as other COVID-19 related health outcomes, due to limited access to healthcare (Cortiula et al., 2020) or loss of health insurance (Blumenthal et al., 1483).

While older populations are, overall, more affected by the direct health effect of COVID-19, younger populations face more indirect effects, such as education disruption (Patheret al., 2020) or even dropout (Alvi & Gupta, 2020), unemployment and financial burden (Inanc, 2020), and uncertainty of the future (Gonzalez et al., 2020). Similarly, young adults were found to have more mental health issues during the pandemic as compared to older adults (Glowacz & Schmits, 2020; Parola et al., 2020). Specifically, a study from the UK observed that young adults experienced high levels of anxiety and depression when the stringent lockdown was introduced (Fancourt et al., 2021). The stringency of government response refers to the measures that are imposed by the government to contain the spread of the virus and it varied across areas and over time (Wijngaards et al., 2020).

Factors such as chronic comorbidities, age, and stringency of government responses may affect the health-related quality of life (HRQoL) and mental well-being of the general population. HRQoL reflects “how well a person functions in their life and his or her perceived wellbeing in physical, mental, and social domains of health” (Stenmanet al., 2010). Measuring HRQoL in the general population is important for assessing health, rationalizing or prioritizing health policies, and evaluating interventions (Guyatt et al., 1993). Mental well-being is defined by the World Health Organization (WHO) as “a state of well-being in which the individual realized his or her own abilities, can cope with the normal stresses of life, can work productively and fruitfully, and is able to make a contribution to his or her community” (Herrman et al., 2005). It records subjective affective responses to daily life, such as positive emotions and life satisfaction (McDowell, 2010). The EQ-5D-5L and World Health Organization-5 Well-Being (WHO-5) are commonly used instruments to measure HRQoL and mental well-being, respectively. While a recent multi-country study showed that strong government response is related to better mental well-being of the general population (Fetzeret al., 2020), it remains to be investigated whether stringency also affects HRQoL.

This study aimed to:

• Assess HRQoL and mental well-being of general population samples of eight countries during the early COVID-19 pandemic in April and May 2020.
• Compare HRQoL and mental well-being of healthy and diseased persons by government response against COVID-19, as measured by the stringency index.
• Examine the relationship between HRQoL and mental well-being and individual characteristics, social position, employment and living situation, chronic disease status, perceptions of being protected against COVID-19 and experience with healthcare.

## 2 Data and methods

### 2.1 Study design and population

In this cross-sectional study, a web-based survey was administered to a cohort of persons from the general population of eight countries: Greece, Italy, the Netherlands, Russia, South Africa, Sweden, the United Kingdom (UK) and the United States of America (US). These countries differed in terms of COVID-19 spread and government measures. Greece, Italy, the Netherlands, Russia, South Africa, Sweden, the United Kingdom (UK) and the United States of America (US).

### 2.2 Data collection procedure and consent

The participants were recruited by an international market research agency that distributed and launched the questionnaire. Existing large internet panels from the eight countries were used, and these samples were designed to be representative of the population aged 18–75 years in each country with regard to age and sex (Appendix Fig. 1). The participants were members of the market research agency's existing voluntary panels. As panel members, the participants had already provided informed consent to participate in online surveys upon registration. Once participating, the data capture system did not allow for missing values. Participants received an incentive in the form of cash or points from the market research company upon completion of the survey. Data were anonymized.

**Fig. 1:**
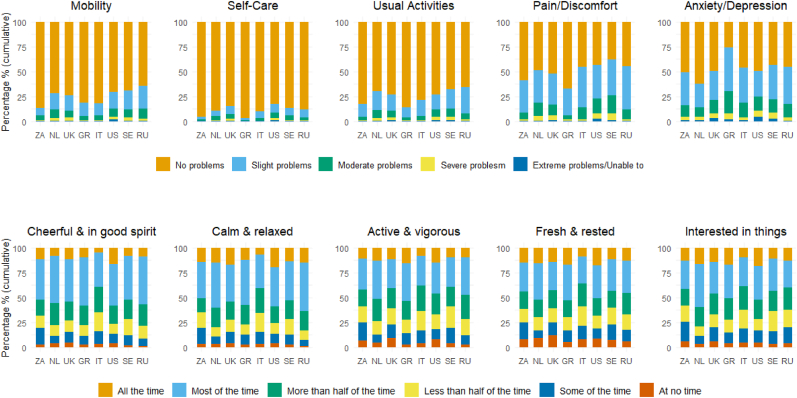
Health domain of the EQ-5D-5L and WHO-5, for eight countries during the early stage of the COVID-19 pandemic (n = 21,354).

### 2.3 Questionnaire

The questionnaire included questions on demographic and social risk factors, health-related and COVID-19-related risk factors, the EQ-5D-5L, and the WHO-5. Data were collected from April 22 to May 5, 2020, in Greece, Italy, the Netherlands, the UK, and the US, and from May 26 to June 1 in Russia, South Africa and Sweden. The questionnaire was translated from English into each country's local language using translation software and then back-translated into English, except when validated translated versions of the instruments were available. Bilingual native speakers verified the translations independently. The questionnaire was translated into Greek, Italian, Dutch (the Netherlands), Russian, English (South Africa), Swedish (Sweden) respectively in Greece, Italy, the Netherlands, Russia, South Africa, and Sweden.

### 2.4 Primary outcome measure

The primary outcome measures were HRQoL (measured by the EQ-5D-5L) and mental well-being (measured by the WHO-5). The EQ-5D-5L measures five domains of HRQoL during a recall period of today (Devlin & Brooks, 2017). It can be used as an indicator of the Burden of Disease such as Quality-Adjusted Life Years (QALY) (Poteet & Craig, 2021). The EQ-5D-5L includes five dimensions: Mobility (MO), Self-Care (SC), Usual activities (UA), Pain/Discomfort (PD), and Anxiety/Depression (AD). The ordinal response options range from “no problems” (“1”) to “extreme problems/unable to (“5”). The EQ-5D-5L index is calculated as a weighted sum of the score of the responses using a value set, which reflects societal preferences for EQ-5D-5L health states. To allow between-country comparisons, the value set from the US (Pickardet al., 2019) was used for each country with the EQ-5D-5L index ranging from below 0 (“worse than death”) to 1(“full health”). The EQ VAS, as part of the EQ-5D-5L instrument, is a self-rated visual analogue scale assessing health state. It ranges from 0 (“the worst imaginable health state”) to 100 (“the best imaginable health state”).

The WHO-5 measures the mental well-being of the past two weeks (Topp et al., 2015). It includes five items: (I have felt) cheerful and in good spirits, calm and relaxed, active and rigorous, I woke up feeling fresh and rested, and my daily life has been filled with things that interest me. The ordinal response options range from “all the time” (“5”) to “at no time” (“0”).

The WHO-5 index is calculated as the unweighted sum of the scores of the responses, multiplied by 4. It ranges from 0 (“worst imaginable well-being”) to 100 (“best imaginable well-being”).

### 2.5 Individual characteristics

The selected factors that may be associated with HRQoL and mental well-being were age, sex, the highest level of education achieved(appendix), occupational status, income, chronic disease status and the number of diseases, smoking status, COVID-19 disease status, living situation, perceptions of being protected against COVID-19, last outpatient visit and experience, and stringency index.

Based on the International Standard Classification of Education (ISCED-2011), the highest level of education achieved was categorized into three groups: up to lower secondary education (ISCED 0, 1 and 2; ‘low’), completed upper secondary education (ISCED 3 and 4; ‘middle’) and tertiary education (ISCED 5 and above; ‘high’).

Two types of data on income were collected: monthly personal income (Greece and Russia) and annual household income (all other countries). Income was categorized into four groups: lower 20% ('low'), middle 60% ('middle'), higher 20% ('high'), and prefer not to answer.

Chronic disease status was measured by the presence of one or more chronic conditions (asthma and chronic bronchitis, severe heart disease, stroke, diabetes, severe back complaints, arthrosis, rheumatism, cancer, memory problems, and/or other problems). The number of chronic conditions was categorized into four groups: “zero”, “one”, “two”, “three”, and “four or more.” Respondents were assumed to be healthy if no chronic conditions were reported and diseased if otherwise. The Last outpatient visit and the experience was based on when this visit occurred and the experience of the access to healthcare received. Two sets of response answers were used for the question on the access to healthcare experienced. The ordinal response option of one set ranges from “very good” to “very bad” while that of the other set ranges from “always good” to “never good.” A random 50% of respondents answered the questionnaire with one of these two sets. The design of the 2-set response answers is part of an experiment on questionnaire wording and is irrelevant to our study. For our study, ordinal response options were merged and ranged from “very good/always good” to “very bad/never good”.

To estimate how strict each government responded to COVID-19 during different time periods, the stringency index at the middle date on which respondents filled out the questionnaire was used for each country. The stringency index was designed by Hale et al., as part of the Oxford COVID-19 Government Response Tracker (OxCGRT) (Hale et al., 2020) and can be used as a proxy of the government responsiveness facing the pandemic. The stringency index ranges from 1 to 100. The higher the index, the stricter ‘lockdown style’ policies that primarily restrict people's behaviours. At the start of the data collection, the stringency index ranged from 64.8 in Sweden and 93.5 in Italy. For easy readership in the figures, we divided the stringency index by 100 and categorized it into five groups with arbitrarily given levels: “Very low” (0.65), “Low” (0.73), “Moderate” (0.78–0.80), “High” (0.84), and “Very high” (0.94).

### 2.6 Statistical analysis

Descriptive analyses were performed for sociodemographic data (age, sex, level of education etc.), EQ-5D-5L dimensions, EQ-5D-5L index, EQ VAS, and WHO-5 items in each country. Simple linear regression was performed on the EQ-5D-5L (rescaled by multiplying by 100) index, EQ VAS and WHO-5 index for risk factors in individual characteristics, social position, employment and living situation, chronic disease status, perceptions of being protected against COVID-19 and experience with healthcare. Variables that had significant or interpretable coefficients were then selected for multiple linear regression analysis. Then, multiple linear regression was performed to examine the relationship between HRQoL and mental well-being and the selected risk factors. Coefficients that reached the minimal important difference (Hoffman et al., 2012; McClure et al., 2017) were considered worthy of interpretation. For both simple and multiple linear regression, respondents (possibly) infected with COVID-19 were excluded and separate analyses were performed among the remaining healthy and diseased respondents. Both regression analyses were first performed for each country and then for all countries pooled. The likelihood ratio test was used for overall p-values where the significance level is set at 0.05. All statistical analyses were carried out using R version 4.0.3.

## 3 Results

### 3.1 Study population

In total, 21,390 respondents completed the survey and 21,354 (99.8%) were included in the study; 2204 persons did not complete the survey (Appendix Table 1). Our sample was representative of the general population by age, sex, and chronic condition status (Appendix) in each country. Table 1 shows the characteristics of the respondents according to country. Overall, the median (IQR) age of the respondents was 44(25). 52% percent of the respondents were female, 53% reported no chronic conditions, 87% were not infected with COVID-19, and 60% felt (very) well protected against COVID-19.

**Table 1:** Sociodemographic background, morbidity status, recent health care experiences and self-reported COVID-19 exposure risk of eight countries during the early stage of COVID-19 pandemic.

		South Africa N = 1067	Netherlands N = 3293	UK N = 3230	Greece N = 959	Italy N = 3210	US N = 3220	Sweden N = 3209	Russia N = 3166	Total N = 21354
Age										
	Median (IQR)	33.0 (20.0)	49.0 (29.0)	44.0 (27.0)	39.0 (20.0)	43.0 (22.0)	46.0 (27.0)	48.0 (28.0)	40.0 (22.8)	44.0(25.0)
	Mean (SD)	36.3 (13.5)	47.9 (16.6)	45.5 (15.9)	40.3 (13.2)	44.0 (14.2)	46.5 (16.1)	47.6 (16.3)	40.7 (14.0)	44.7 (15.7)
Age group										
	18–24 yrs.	235 (22.0%)	338 (10.3%)	315 (9.8%)	151 (15.7%)	276 (8.6%)	355 (11.0%)	301 (9.4%)	531 (16.8%)	2502 (11.7%)
	25–34 yrs.	343 (32.1%)	520 (15.8%)	649 (20.1%)	206 (21.5%)	626 (19.5%)	539 (16.7%)	536 (16.7%)	704 (22.2%)	4123 (19.3%)
	35–44 yrs.	201 (18.8%)	539 (16.4%)	708 (21.9%)	229 (23.9%)	827 (25.8%)	608 (18.9%)	556 (17.3%)	603 (19.0%)	4271 (20.0%)
	45–54 yrs.	149 (14.0%)	577 (17.5%)	510 (15.8%)	203 (21.2%)	675 (21.0%)	592 (18.4%)	616 (19.2%)	680 (21.5%)	4002 (18.7%)
	55–64 yrs.	98 (9.2%)	636 (19.3%)	511 (15.8%)	137 (14.3%)	463 (14.4%)	558 (17.3%)	551 (17.2%)	488 (15.4%)	3442 (16.1%)
	65–75 yrs.	41 (3.8%)	683 (20.7%)	537 (16.6%)	33 (3.4%)	343 (10.7%)	568 (17.6%)	649 (20.2%)	160 (5.1%)	3014 (14.1%)
Sex										
	Male	488 (45.7%)	1587 (48.2%)	1558 (48.2%)	457 (47.7%)	1537 (47.9%)	1414 (43.9%)	1519 (47.3%)	1588 (50.2%)	10148 (47.5%)
	Female	579 (54.3%)	1706 (51.8%)	1672 (51.8%)	502 (52.3%)	1673 (52.1%)	1806 (56.1%)	1690 (52.7%)	1578 (49.8%)	11206 (52.5%)
Education level										
	High	656 (61.5%)	1462 (44.4%)	1975 (61.1%)	587 (61.2%)	1333 (41.5%)	1855 (57.6%)	1884 (58.7%)	1645 (52.0%)	11397 (53.4%)
	Middle	406 (38.1%)	1000 (30.4%)	1180 (36.5%)	335 (34.9%)	1429 (44.5%)	1142 (35.5%)	988 (30.8%)	1460 (46.1%)	7940 (37.2%)
	Low	5 (0.5%)	831 (25.2%)	75 (2.3%)	37 (3.9%)	448 (14.0%)	223 (6.9%)	337 (10.5%)	61 (1.9%)	2017 (9.4%)
Occupation										
	Employed	656 (61.5%)	1684 (51.1%)	1932 (59.8%)	508 (53.0%)	1848 (57.6%)	1671 (51.9%)	1574 (49.0%)	2006 (63.4%)	11879 (55.6%)
	Student	131 (12.3%)	245 (7.4%)	100 (3.1%)	91 (9.5%)	223 (6.9%)	98 (3.0%)	232 (7.2%)	254 (8.0%)	1374 (6.4%)
	Unemployed	173 (16.2%)	379 (11.5%)	394 (12.2%)	267 (27.8%)	726 (22.6%)	526 (16.3%)	445 (13.9%)	477 (15.1%)	3387 (15.9%)
	Retired	47 (4.4%)	651 (19.8%)	575 (17.8%)	77 (8.0%)	385 (12.0%)	665 (20.7%)	783 (24.4%)	382 (12.1%)	3565 (16.7%)
	Unable to work	60 (5.6%)	334 (10.1%)	229 (7.1%)	16 (1.7%)	28 (0.9%)	260 (8.1%)	175 (5.5%)	47 (1.5%)	1149 (5.4%)
Income										
	High	271 (25.4%)	805 (24.4%)	650 (20.1%)	302 (31.5%)	413 (12.9%)	909 (28.2%)	683 (21.3%)	473 (14.9%)	4506 (21.1%)
	Middle	551 (51.6%)	1383 (42.0%)	1537 (47.6%)	281 (29.3%)	1760 (54.8%)	1589 (49.3%)	1553 (48.4%)	2024 (63.9%)	10678 (50.0%)
	Low	172 (16.1%)	547 (16.6%)	773 (23.9%)	274 (28.6%)	691 (21.5%)	546 (17.0%)	596 (18.6%)	446 (14.1%)	4045 (18.9%)
	Unwilling to tell	73 (6.8%)	558 (16.9%)	270 (8.4%)	102 (10.6%)	346 (10.8%)	176 (5.5%)	377 (11.7%)	223 (7.0%)	2125 (10.0%)
Chronic conditions										
	0	1828 (56.6%)	1601 (49.7%)	1639 (49.8%)	1983 (61.8%)	570 (59.4%)	1761 (55.6%)	615 (57.6%)	1421 (44.3%)	11418 (53.5%)
	1	882 (27.3%)	986 (30.6%)	1026 (31.2%)	857 (26.7%)	300 (31.3%)	948 (29.9%)	310 (29.1%)	1047 (32.6%)	6356 (29.8%)
	2	325 (10.1%)	339 (10.5%)	368 (11.2%)	236 (7.4%)	62 (6.5%)	300 (9.5%)	91 (8.5%)	453 (14.1%)	2174 (10.2%)
	3	125 (3.9%)	171 (5.3%)	166 (5.0%)	74 (2.3%)	16 (1.7%)	102 (3.2%)	32 (3.0%)	177 (5.5%)	863 (4.0%)
	4 or more	70 (2.2%)	123 (3.8%)	94 (2.9%)	60 (1.9%)	11 (1.1%)	55 (1.7%)	19 (1.8%)	111 (3.5%)	543 (2.5%)
Smoking										
	No	2930 (90.7%)	2921 (90.7%)	2977 (90.4%)	2688 (83.7%)	761 (79.4%)	2676 (84.5%)	892 (83.6%)	2868 (89.4%)	18713 (87.6%)
	Yes	300 (9.3%)	299 (9.3%)	316 (9.6%)	522 (16.3%)	198 (20.6%)	490 (15.5%)	175 (16.4%)	341 (10.6%)	2641 (12.4%)
COVID-19 status										
	Not infected	2745 (85.0%)	2704 (84.0%)	2834 (86.1%)	2879 (89.7%)	897 (93.5%)	2963 (93.6%)	994 (93.2%)	2514 (78.3%)	18530 (86.8%)
	Infected but recovered	30 (0.9%)	46 (1.4%)	28 (0.9%)	16 (0.5%)	1 (0.1%)	17 (0.5%)	3 (0.3%)	37 (1.2%)	178 (0.8%)
	Maybe infected	444 (13.7%)	426 (13.2%)	420 (12.8%)	315 (9.8%)	60 (6.3%)	182 (5.7%)	70 (6.6%)	647 (20.2%)	2564 (12.0%)
	Infected and not recovered	11 (0.3%)	44 (1.4%)	11 (0.3%)	0 (0.0%)	1 (0.1%)	4 (0.1%)	0 (0.0%)	11 (0.3%)	82 (0.4%)
Living situation										
	Living alone	638 (19.8%)	714 (22.2%)	871 (26.5%)	328 (10.2%)	131 (13.7%)	398 (12.6%)	109 (10.2%)	1010 (31.5%)	4199 (19.7%)
	Living alone with children	277 (8.6%)	255 (7.9%)	216 (6.6%)	190 (5.9%)	70 (7.3%)	201 (6.3%)	120 (11.2%)	290 (9.0%)	1619 (7.6%)
	Living with other adults	1321 (40.9%)	1277 (39.7%)	1318 (40.0%)	1326 (41.3%)	371 (38.7%)	1132 (35.8%)	283 (26.5%)	1123 (35.0%)	8151 (38.2%)
	Living with other adults and children	919 (28.5%)	836 (26.0%)	834 (25.3%)	1308 (40.7%)	368 (38.4%)	1284 (40.6%)	496 (46.5%)	720 (22.4%)	6765 (31.7%)
	Other	75 (2.3%)	138 (4.3%)	54 (1.6%)	58 (1.8%)	19 (2.0%)	151 (4.8%)	59 (5.5%)	66 (2.1%)	620 (2.9%)
Feeling protected										
	Very well	737 (22.8%)	944 (29.3%)	457 (13.9%)	486 (15.1%)	395 (41.2%)	428 (13.5%)	312 (29.2%)	512 (16.0%)	4271 (20.0%)
	Well	1231 (38.1%)	1101 (34.2%)	1705 (51.8%)	1409 (43.9%)	397 (41.4%)	1202 (38.0%)	389 (36.5%)	1133 (35.3%)	8567 (40.1%)
	Reasonably	1096 (33.9%)	990 (30.7%)	976 (29.6%)	1111 (34.6%)	140 (14.6%)	1132 (35.8%)	323 (30.3%)	1117 (34.8%)	6885 (32.2%)
	Insufficiently	166 (5.1%)	185 (5.7%)	155 (4.7%)	204 (6.4%)	27 (2.8%)	404 (12.8%)	43 (4.0%)	447 (13.9%)	1631 (7.6%)
Last outpatient visit										
	>3 months ago	2228 (69.0%)	1909 (59.3%)	2159 (65.6%)	2060 (64.2%)	641 (66.8%)	2364 (74.7%)	702 (65.8%)	1947 (60.7%)	14010 (65.6%)
	1–3 months ago	673 (20.8%)	758 (23.5%)	706 (21.4%)	846 (26.4%)	220 (22.9%)	467 (14.8%)	220 (20.6%)	532 (16.6%)	4422 (20.7%)
	1–4 weeks ago	215 (6.7%)	328 (10.2%)	241 (7.3%)	193 (6.0%)	65 (6.8%)	182 (5.7%)	109 (10.2%)	426 (13.3%)	1759 (8.2%)
	Last week	114 (3.5%)	225 (7.0%)	187 (5.7%)	111 (3.5%)	33 (3.4%)	153 (4.8%)	36 (3.4%)	304 (9.5%)	1163 (5.4%)
Access of healthcare										
	Very good/Always good	1087 (33.7%)	1632 (50.7%)	1264 (38.4%)	957 (29.8%)	301 (31.4%)	603 (19.0%)	397 (37.2%)	979 (30.5%)	7220 (33.8%)
	Good/Usually good	1264 (39.1%)	1052 (32.7%)	1501 (45.6%)	1457 (45.4%)	366 (38.2%)	1304 (41.2%)	403 (37.8%)	1201 (37.4%)	8548 (40.0%)
	Fair/Sometimes good	668 (20.7%)	421 (13.1%)	414 (12.6%)	625 (19.5%)	188 (19.6%)	777 (24.5%)	177 (16.6%)	731 (22.8%)	4001 (18.7%)
	Bad/Usually not good	163 (5.0%)	84 (2.6%)	95 (2.9%)	134 (4.2%)	79 (8.2%)	337 (10.6%)	66 (6.2%)	224 (7.0%)	1182 (5.5%)
	Very bad/Never good	48 (1.5%)	31 (1.0%)	19 (0.6%)	37 (1.2%)	25 (2.6%)	145 (4.6%)	24 (2.2%)	74 (2.3%)	403 (1.9%)

Note to Table 1: For Greece and Russia, income represents individual monthly income, for the rest, it represents annual household income. Smoking is referring to the self-perception of risk to COVID-19 due to smoking. The order of the country in the table is by median EQ-5D-5L index score.

### 3.2 Prevalence of HRQoL and mental well-being

**Fig. 2:**
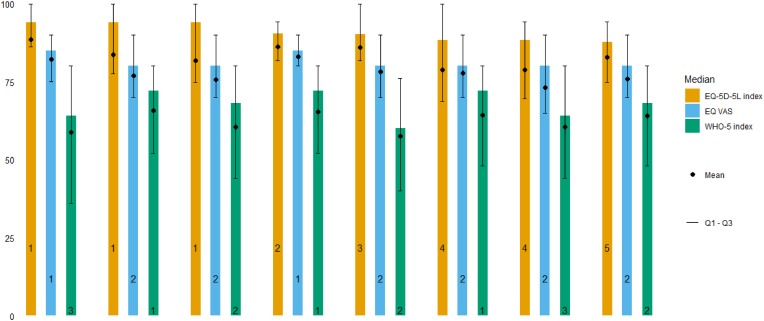
HRQoL and mental well-being (EQ-5D-5L index, EQ VAS and WHO-5 index) in eight countries during the early stage of the COVID-19 pandemic (n = 21,354) Note to Fig. 2: EQ-5D-5L value set of the US is chosen for all countries to allow comparison across countries. The number in the figure refers to the ranking by country in each outcome.

Fig. 1, Fig. 2 illustrate the distribution of EQ-5D-5L and WHO-5 domains and indices by country. In terms of the median EQ-5D-5L index, South Africa ranked the highest, and Russia ranked the lowest. For the median EQ VAS, South Africa ranked the highest. With regard to the median WHO-5 index, the Netherlands ranked the highest and South Africa ranked the lowest.

**Fig. 3:**
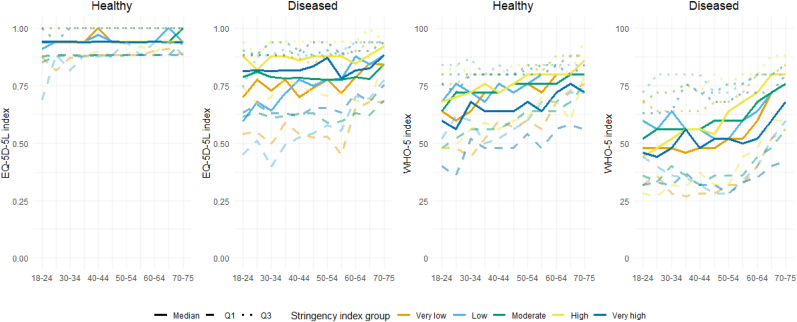
EQ-5D-5L index and WHO-5 according to age categories and stringency of government response, for healthy (N = 11,418) and diseased (N = 9936) persons separately.

The stringency index ranged from 64.8 in Sweden and 93.5 in Italy (Appendix Table 2). Fig. 3 presents the distribution of EQ-5D-5L and WHO-5 index scores according to stringency category by age groups and chronic disease status. Overall, diseased respondents had lower EQ-5D-5L index and WHO-5 scores compared to healthy respondents. Younger respondents had lower WHO-5 scores compared to older respondents. This pattern was more profound among diseased than healthy respondents. For the EQ-5D-5L, this pattern was found only among the diseased respondents for certain stringency levels. Additionally, in countries where stringency was high, diseased respondents had higher EQ-5D-5L index scores compared to countries where stringency was lower. This pattern was not observed for the WHO-5 index.

**Fig. 4:**
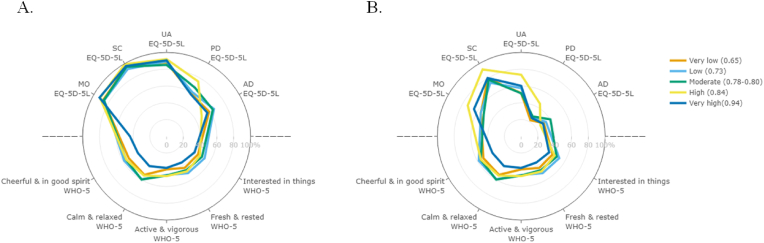
Optimal health domains of HRQoL and mental well-being according to the stringency of government response among healthy (A, N = 11,418) and diseased (B, N = 9936) persons separatelyNote to Fig. 4: optimal health refers to “no problems” for EQ-5D-5L and “at no time” for WHO-5.

Fig. 4 presents the prevalence of optimal health in each domain for EQ-5D-5L and WHO-5 according to the stringency index. Among healthy respondents, the prevalence of perfect mental well-being in WHO-5 domains decreased with increasing higher stringency. Among diseased respondents, the prevalence of perfect health for EQ-5D-5L domains increased and the prevalence of perfect mental well-being in WHO-5 domains decreased with increasing stringency.

### 3.3 Association between HRQoL and mental well-being and other risk factors

From the simple linear regression analysis, almost all risk factors were selected for multiple linear regression with the exception of last outpatient visit due to difficulty in interpretation (Appendix). Table 2 shows the results of the multiple linear regression analysis among healthy and diseased respondents, excluding respondents infected with COVID-19. Overall, almost all groups had a lower score with the HRQoL and mental well-being compared to the reference group. Stringency had a positive effect on the EQ-5D-5L index and EQ VAS and a negative effect on WHO-5. Among healthy respondents, being unable to work had the worst effect on the EQ-5D-5L index, while feeling insufficiently protected against COVID-19 had the worst effect on the EQ VAS and WHO-5 index. Among diseased respondents, having more than four chronic conditions had the worst effect on both the EQ-5D-5L and EQ VAS and feeling insufficiently protected had the worst effect on the WHO-5. Several factors reached minimum important difference in terms of mean EQ-5D-5L, including unable to work, feeling insufficiently protected against COVID-19, (very) bad experience with the access of healthcare received, and having two or more chronic conditions. There is no available minimum important difference yet for the WHO-5 index, but the same factors stood out with the largest difference compared to the reference group.

**Table 2:** Multiple linear regression on EQ-5D-5L and WHO-5 according to non-health and health-related risk factors during the early stage of the COVID-19 pandemic, for healthy (N = 10,411) and diseased (N = 8119) persons separately, excluding persons infected with COVID-19.

		Healthy			Diseased		
		N = 10411			N = 8119		
		EQ-5D-5L index	EQ VAS	WHO-5 index	EQ-5D-5L index	EQ VAS	WHO-5 index
	(Intercept)	92.7	89.7	106.9	81.6	80.2	89.0
Age group		*		*		*	*
	25–34 yrs.	2.1	0.1	1.0	0.1	−0.9	0.1
	35–44 yrs.	2.0	−0.1	1.3	0.0	−1.4	1.6
	45–54 yrs.	2.4	−0.4	3.7	0.7	−2.2	3.8
	55–64 yrs.	2.0	−0.4	4.8	1.2	−1.0	7.4
	65–75 yrs.	2.5	0.0	6.4	2.8	0.1	10.0
Sex				*		*	*
	Female	−0.4	0.2	−4.5	−0.8	1.0	−5.9
Education level				*	*	*	*
	Middle	−0.1	−0.1	1.1	−1.2	−1.3	0.8
	Low	0.2	0.2	2.1	0.8	0.4	3.8
Occupation		*	*	*	*	*	*
	Student	−0.8	−0.7	−5.5	−1.1	−0.6	−5.9
	Unemployed	−1.7	−1.1	−4.3	−3.1	−1.6	−6.0
	Retired	−0.6	0.3	1.0	−4.4	−3.3	−0.1
	Unable to work	−7.9	−5.0	−10.6	−22.5	−12.4	−11.3
Income		*	*	*	*	*	*
	Middle	−1.1	−1.3	−1.3	−2.1	−2.0	−3.1
	Low	−1.4	−1.5	−1.9	−4.3	−4.3	−5.8
	Unwilling to tell	−0.2	0.0	0.2	−1.2	−1.5	−2.7
Living situation		*				*	*
	Living alone with children	−0.7	−0.5	1.1	1.0	0.3	2.4
	Living with other adults	0.0	0.3	0.8	0.9	1.7	1.9
	Living with other adults and children	0.0	0.2	1.5	0.5	1.9	2.9
	Other	−2.3	0.0	−1.0	−0.2	1.3	0.0
Smoking		*	*	*	*		*
	Yes	−2.4	−1.5	−3.1	−2.2	−0.8	−4.4
Chronic conditions					*	*	*
	2	\	\	\	−8.9	−6.1	−6.2
	3	\	\	\	−17.2	−11.1	−10.7
	4	\	\	\	−29.6	−16.7	−16.8
Feeling protected against COVID-19		*	*	*	*	*	*
	Well	−1.1	−4.4	−7.9	−0.5	−3.2	−5.9
	Reasonably	−2.3	−7.4	−14.7	−4.1	−8.3	−14.0
	Insufficiently	−5.1	−8.9	−20.7	−8.3	−11.5	−19.1
Access of healthcare		*	*	*	*	*	*
	Good/Usually good	−1.8	−3.1	−5.6	−2.5	−2.4	−5.1
	Fair/Sometimes good	−3.8	−5.9	−10.4	−6.2	−5.5	−9.9
	Bad/Usually not good	−6.1	−5.4	−11.4	−9.1	−5.8	−13.3
	Very bad/Never good	−4.1	−4.3	−12.7	−12.9	−7.3	−15.2
Stringency index		*		*	*	*	*
		3.5	2.7	−30.6	12.3	7.4	−17.9

Note to Table 2: Multiple linear regression on 100*EQ-5D-5L, EQ-VAS, and WHO-5 (scale 0–100). Asterisk (*) refer to the categories that are statistically significant (<0.05). Reference group: 18–24 yrs, male, high educated, being employed, high household income, living alone, no extra risk of COVID-19 from smoking, 1 chronic condition (for diseased respondents), feeling very well protected against COVID-19, very good experience with the access to healthcare. For healthy respondents, chronic conditions were not included in the model. Therefore, in the table shown as “\”. The numeric stringency index was used and divided by 100.

Noticeable between-country pattern differences were also found; multiple linear regression results can be found in Appendix Tables 3 and 4.

## 4 Discussion

### 4.1 HRQoL and mental well-being

The general populations of the eight countries included in our study experienced the first wave of COVID-19 during the end of April and early May 2020. The governments for these countries expressed a range of political and macroeconomic points of view, in addition to variation in the stringency of governmental responses. However, we observed some similarities between countries with respect to the health impact on healthy and diseased respondents. Compared to healthy respondents, diseased respondents scored lower on all EQ-5D-5L and WHO-5 domains, resulting in worse HRQoL and mental well-being.

In addition, we found that stringency of government response had opposite patterns for HRQoL and mental well-being, namely a small positive relation with HRQoL and a strong negative relation with mental well-being, although the magnitude of effects differed for healthy persons and diseased persons. Furthermore, the effect of the stringency of government response was most favourable for diseased respondents, because the magnitude of positive relation is larger and of negative relation is smaller, compared to healthy respondents.

For mental well-being, we suspect that the more stringent the government response, the higher burden is projected onto the citizens. A more stringent government response results in a visible decline in new infections (Fang et al., 2020). Such a response could lead to higher trust towards the government and other citizens and, ultimately, a higher sense of protection and security (Rieger and WangAbgerufen von, 2020). However, a more stringent response also means more strict movement restrictions or confinement, social isolation, and a prolonged pause of major life events. It could lead to fear and uncertainty. Some studies confirmed our findings. O'Hara et al. found an increase in worry and depression with an increase in stringency (O'Hara et al., 2020). Lee et al. revealed higher depression in countries with a higher stringency index (Lee et al., 2021). However, a few other studies established different results. Fetzer *et al* (Fetzeret al., 2020) and Kim *et al* (Kim & Jung, 2021) found stricter government response associated with lower distress. However, the study by Fetzer et al. included respondents from more than 50 countries and investigated the effect of subjectively perceived stringency by the respondents rather than the stringency index of the COVID-19 Government Response Tracker that is based on nine objective response indicators.

### 4.2 Risk factors

Regression coefficients were largest for mental well-being compared to HRQoL, and almost all groups had a negative relationship with HRQoL and mental well-being compared to the reference group. Several studies have established that feeling protected against COVID-19 is negatively associated with anxiety (Monterrosa-Castro et al., 2020a), discrimination (Monterrosa-Castro et al., 2020b) and post-traumatic stress symptoms (Asaoka et al., 2020) among medical staff and general populations. Our study further confirmed that feeling protected against COVID-19 is associated with better mental well-being. Feeling protected may reduce the perception of risk, where being perceived as at risk has been negatively associated with mental well-being (Krok & Zarzycka, 2020).

We observed an age gradient for mental well-being where younger respondents had worse and older respondents surprisingly better mental well-being. This gradient was also confirmed by regression coefficients and was most pronounced in the diseased subgroup. These findings are consistent with the majority of other studies indicating that younger adults were mentally affected by the pandemic to a greater degree than other age groups (Barber & Kim, 2021; Liu et al., 2020; Pieh et al., 2020). The finding that mental well-being is lower in younger adults may be due to a lower tolerance for uncertainty among younger adults (Basevitz et al., 2008), where uncertainty pertains to education, career and social life. Cohort effects (Clark, 2019, pp. 387–408) and selection bias may also have contributed to the gradient.

### 4.3 Strengths and limitations

Our study collected data from respondents from eight countries at the early stage of the pandemic. These eight countries included political heterogeneity, different systems of public health, and variation in stringency.

There are some limitations to this study. First, chronic conditions were self-reported. We assumed respondents accurately reported all of their conditions, but this could not be verified. Second, at the time of sampling mass testing for COVID-19 was not available in the general population and even was restricted at health care facilities. Therefore, we were unable to distinguish with certainty between respondents who were infected with COVID-19 and respondents who were not. Partly due to that, we excluded persons with possible COVID-19 infection from the regression analyses. Third, given that the questionnaire was administered with only one main national language in each country, we may have lost representativeness of the population that speaks another language or who are not literate, and these groups might have significantly different health states due to their minority position. Finally, younger males were under-represented in our sample. As a result, our findings may have underestimated true findings, because younger respondents reported worse mental well-being compared to other age groups. Additionally, our findings may have underestimated educational differences in terms of HRQoL and mental well-being due to the under-representation of persons with lower levels of educational attainment. It is also worth noticing that we chose the value set of the US to calculate the EQ-5D-5L index because it is driven from a relatively new study in a large country that contains groups that differ according to a number of factors, such as race and ethnicity. Using a different value set may result in different findings.

## 5 Conclusions

The stringency of government response had differing patterns for HRQoL and mental well-being with a small positive relation with HRQoL and strong negative relation with mental well-being. The magnitude of effects differed for healthy and diseased persons and by age but was most favourable for diseased and older persons. Specific responses to the mental well-being of the general population should be considered when designing interventions against COVID-19. Moreover, understanding how HRQoL and mental well-being change over time will be critical as the pandemic continues to evolve and various countries have experienced different trajectories in terms of government responses and the availability of vaccines. Therefore, follow-up studies should be conducted and disseminated.

## Funding

This study was funded by the 10.13039/501100006419EuroQol Research Foundation (grant number 77-2020-RA).

## Availability of data and material

The dataset used and analysed during the current study is available from the senior author on reasonable request.

## Code availability

Available on request.

## Authors' contributions

All authors contributed to the conception and design of the study. JH, GB, and MJ collected the data. Material preparation analysis and interpretation of data were performed by DL and JH. DL wrote the first draft of the manuscript. All authors reviewed and critically revised the manuscript. All authors read and approved the final manuscript as submitted and agree to be accountable for all aspects of the work.

## Ethical approval

Ethical approval was obtained from the Erasmus MC ethics review board (approval MEC-2020-0266). Data were collected anonymously. Individual consent was obtained from all participants before filling out the questionnaire.

## Declaration of competing interest

The authors declare that they have no conflict of interest.
