# Supplementary material for: Health-related quality of life and mental well-being of healthy and diseased persons in 8 countries: Does stringency of government response against early COVID-19 matter?
Source: SSM Popul Health. 2021 Sep 1;15:100913. doi: 10.1016/j.ssmph.2021.100913 (PMC8426285; doi:10.1016/j.ssmph.2021.100913)
Supplement: Multimedia component 1 [file mmc1.docx]

# Health-related quality of life and mental well-being of healthy and diseased persons in 8 countries: does stringency of government response against early COVID-19 matter?

Di Long^1^, Juanita A. Haagsma^1^, Mathieu F. Janssen^2^, John N. Yfantopoulos^3^, Erica I. Lubetkin^4^ Gouke J. Bonsel^1,5^

1. Department of Public health, Erasmus MC, Rotterdam, The Netherlands

2. Section Medical Psychology and Psychotherapy, Department of Psychiatry, Erasmus MC, Rotterdam, The Netherlands

3. MBA - Health, Department of Economics, National and Kapodistrian University of Athens, Athens, Greece

4. Department of Community Health and Social Medicine, CUNY School of Medicine, New York, NY, USA

5. EuroQol Research Foundation, Rotterdam, the Netherlands

Corresponding author:

Di Long, Department of Public health, Erasmus MC, Rotterdam, The Netherlands; address: Doctor Molewaterplein 40, 3015 GD; P.O. Box 2040, 3000 CA; email: [d.long@erasmusmc.nl](mailto:d.long@erasmusmc.nl)

# Appendix

### List of table and figures

Figure 1A-H: Distribution of sex and age categories of national population and of our sample, by country

Table 1: Percentage distribution of age, sex, and level of education among study dropouts as compared to respondents

Table 2: Distribution of stringency index by country during data collection dates

Table 3: Multiple linear regression on HRQoL and mental well-being according to non-health and health-related risk factors during the early stage of the COVID-19 pandemic, for healthy (N=10,411) respondents in each country, excluding persons infected with COVID-19

Table 4: Multiple linear regression on HRQoL and mental well-being according to non-health and health-related risk factors during the early stage of the COVID-19 pandemic, for diseased (N=8,119) respondents in each country, excluding persons infected with COVID-19

### *Stringency index by country*

Stringency index, developed by Oxford COVID-19 Government Response Tracker (OxCGRT), ranges from 1 to 100. At the start of the data collection, the stringency index ranged from 64.8 in Sweden and 93.5 in Italy. Table 1 shows the distribution of stringency index by country during the dates of data collection.

### *Data representativeness*

To measure to what extent our data represent national data on age and sex, the set of figures 1A-H were constructed. Respondents aged 18-, 19- and 75-years were excluded from the figures.

The percentage represents the percentage of that sex-age category out of the total population aged 20-74.

Overall, data representation is good. However, we observed that younger age males (20-29 years old) were underrepresented in many countries. Moreover, in Greece, older females were underrepresented while younger females were overrepresented.

Our data also had good representation on the percentage of people with any chronic conditions. Estimation of national percentage of people living with chronic conditions from OECD and other sources[1, 2] showed similar percentage as out data.

### *Dropout characteristics*

2204 people started but did not finish the questionnaire (Table 2). Missing in the table means dropping out prior to completing this questionnaire.

Compared to completers, significant difference is marked by *. Dropouts were significantly younger in Russia, more likely to be female in Sweden and Russia, and differently distributed in level of education in all countries.

### *Simple linear regression results in each country*

Simple linear regressions were performed in each country on HRQoL and mental well-being for age, sex, level of education, income, living situation, perceived risk of COVID-19 due to smoking, feeling protected against COVID-19, last outpatient visit and experience with access of healthcare. Almost all coefficients of the risk factors were significant and should be considered in the multiple linear regression. However, we had difficulty in interpreting the variable on last outpatient visit. Therefore, we excluded it from the multiple linear regression model.

### *Multiple linear regression results in each country*

Table 3 and Table 4 presents the results from multiple linear regression analysis for each country among healthy (Table 3) and diseased (Table 4) groups.

For HRQoL among healthy respondents, several distinguishing differences were noted between countries. The US had the largest significantly positive association between older age groups and EQ-5D-5L index. Sex was significantly negatively associated with EQ-5D-5L index in Greece and Russia and negatively associated with EQ VAS in South Africa but positively associated with EQ VAS in the UK. Income was only significantly associated with EQ-5D-5L index in Russia, and significantly associated with EQ VAS in South Africa, the UK and the US. Feeling protected was not significantly associated with EQ-5D-5L index in South Africa and the Netherlands. For mental well-being among healthy respondents, South Africa, the UK, and Greece did not have strong associations between age and the WHO-5 index, and only South Africa and Greece had a strong association between living situation and the WHO-5 index.

For HRQoL among diseased respondents, the Netherlands and the US had significantly positive associations between age and the EQ-5D-5L index, Russia had significantly negative associations. Sweden had significantly positive associations between age and EQ VAS, while the Netherlands and Russia had significantly negative associations. Sex was only significantly associated with EQ-5D-5L index in the Netherlands, Sweden, and Russia, and with EQ VAS in Russia. Education was only significantly associated with EQ-5D-5L index in the UK. Income was only significantly associated with EQ-5D-5L index in South Africa, the UK and the US, and with EQ VAS in the UK and the US. Smoking was only significantly associated with EQ-5D-5L in Italy (index) and Greece (EQ VAS). For mental well-being among diseased respondents, age groups were not significantly associated with WHO-5 index in South Africa, the UK, and Italy. Education and Living situation were only significantly associated with WHO-5 index in South Africa.

### *Figures and tables*

#### Figure 1(A-H). Distribution of sex and age categories of national population and of our sample, by country


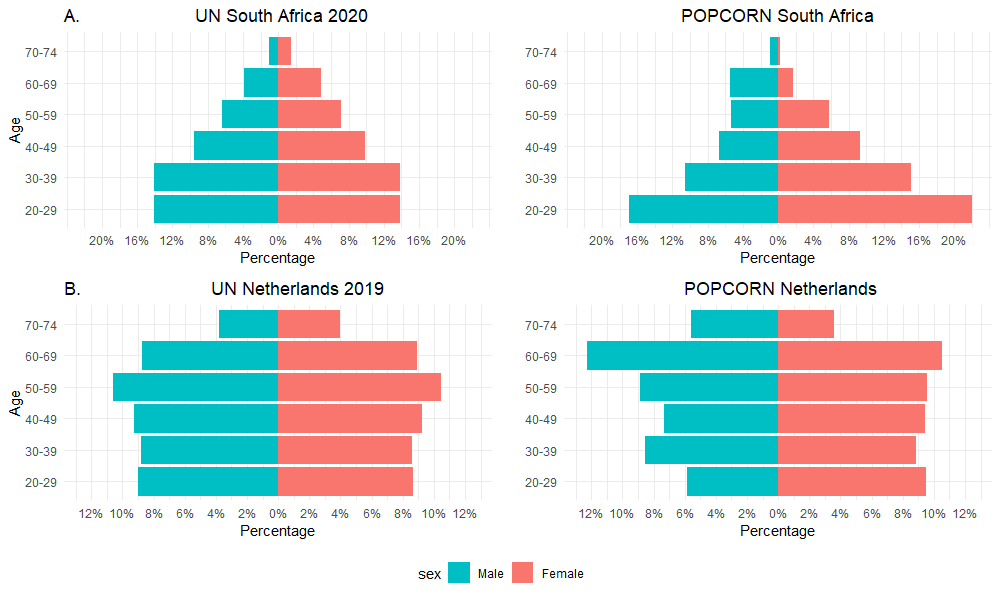


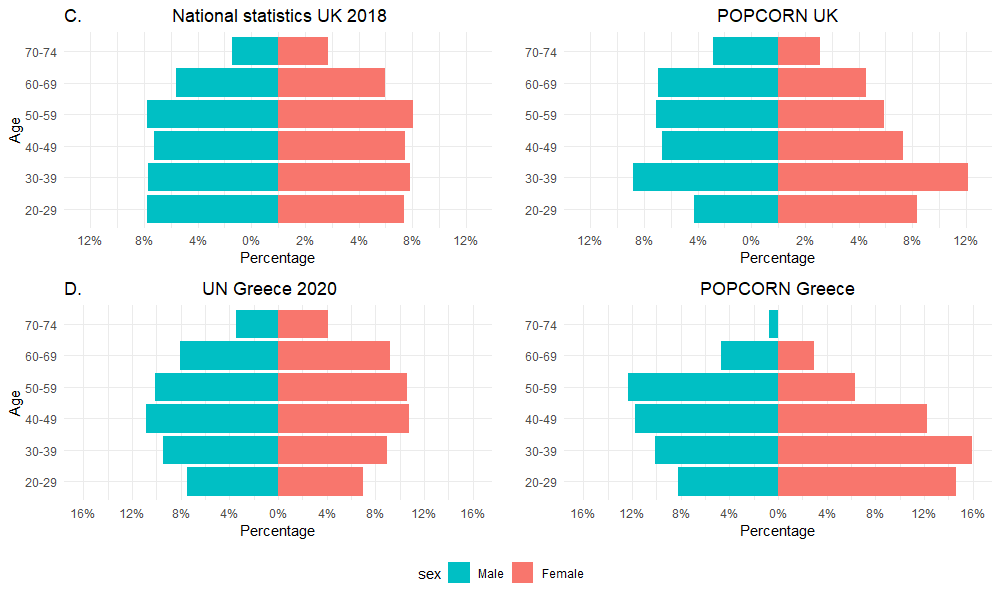

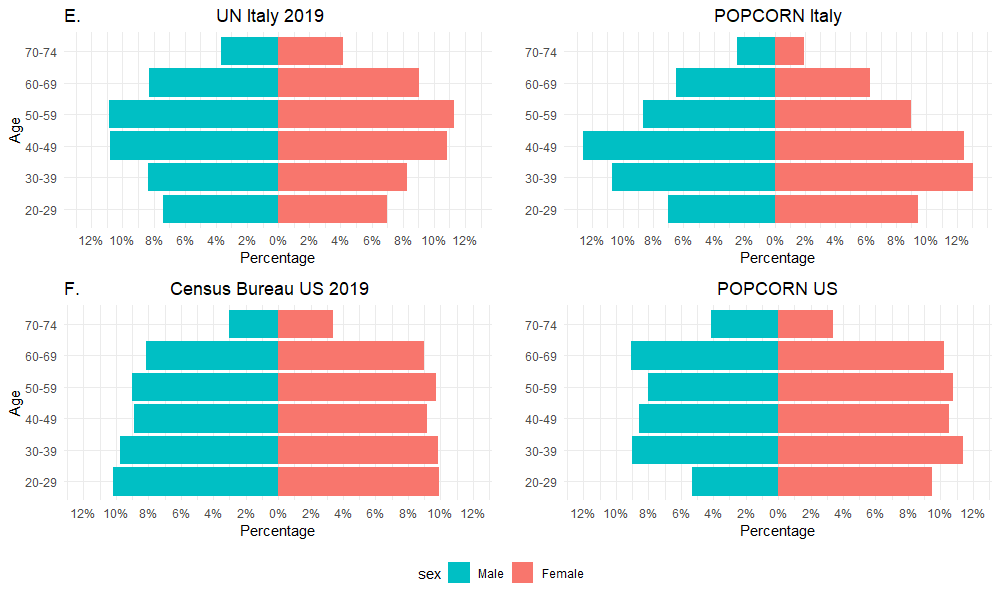

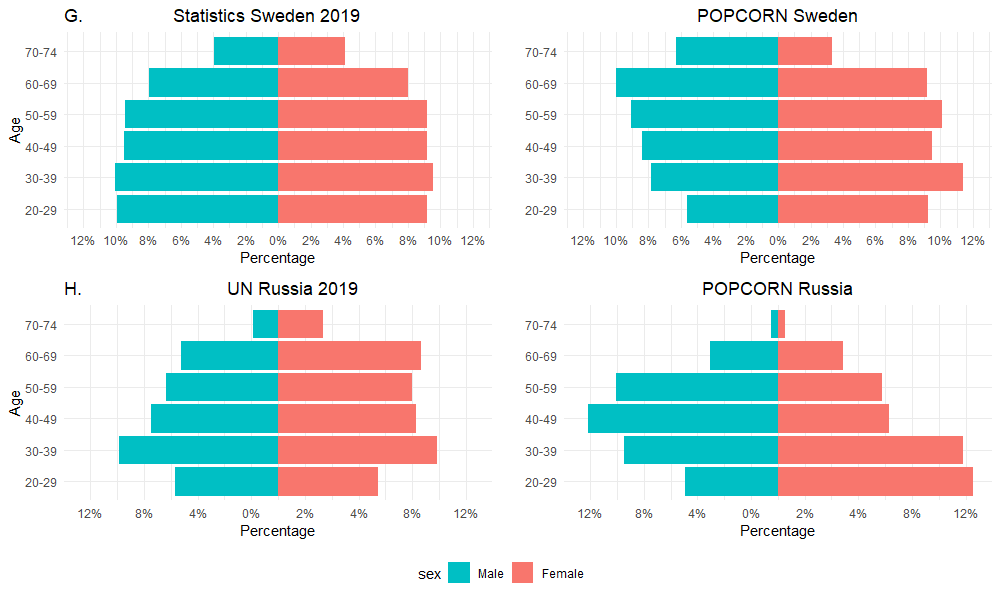


#### Table 1. Percentage distribution of age, sex, and level of education among dropouts, compared to respondents in our study

|  |  | South Africa % | | Netherlands % | | UK % | | Greece % | |
| --- | --- | --- | --- | --- | --- | --- | --- | --- | --- |
|  |  | ***Dropout***  ***N=97*** | ***Completer***  ***N=1067*** | ***Dropout***  ***N=489*** | ***Completer***  ***N=3293*** | ***Dropout***  ***N=354*** | ***Completer***  ***N=3230*** | ***Dropout***  ***N=85*** | ***Completer***  ***N=959*** |
| % |  | 8.3% |  | 12.9% |  | 9.9% |  | 8.1% |  |
| Age | |  |  |  |  |  |  |  |  |
|  | Median (IQR) | 30.0 (30.0) | 33 (20) | 50.0 (31.0) | 49.0 (29.0) | 45.0 (29.0) | 44.0(27.0) | 39.0 (21.0) | 39.0 (20.0) |
|  | Mean (SD) | 33.9 (13.7) | 36.3 (13.5) | 48.4 (17.3) | 47.8 (16.6) | 45.8 (16.8) | 45.5 (15.9) | 39.1 (12.9) | 40.3 (13.2) |
| Sex | |  |  |  |  |  |  |  | |
|  | Male | 47.4% | 45.7% | 44.6% | 48.2% | 44.1% | 48.2% | 44.7% | 47.7% |
|  | Female | 52.6% | 54.3% | 55.4% | 51.8% | 55.9% | 51.8% | 55.3% | 52.3% |
| Education | | * | | * | | * | | * | |
|  | High | 76.3% | 61.5% | 53.6% | 44.4% | 43.2% | 61.1% | 45.9% | 61.2% |
|  | Middle | 12.4% | 38.1% | 26.4% | 30.4% | 48.9% | 36.5% | 50.6% | 34.9% |
|  | Low | 11.3% | 0.5% | 19.6% | 25.2% | 7.9% | 2.3% | 3.5% | 3.9% |
|  |  | ***Italy %*** | | ***US %*** | | ***Sweden %*** | | ***Russia %*** | |
|  |  | ***Dropout***  ***N=144*** | ***Completer***  ***N=3210*** | ***Dropout***  ***N=359*** | ***Completer***  ***N=3220*** | ***Dropout***  ***N=442*** | ***Completer***  ***N=3209*** | ***Dropout***  ***N=234*** | ***Completer***  ***N=3166*** |
| % |  | 4.3% |  | 10.0% |  | 12.2% |  | 6.9% |  |
| Age | |  | |  | |  | | * | |
|  | Median (IQR) | 42.0 (24.3) | 43.0 (22.0) | 46.0 (29.0) | 46.0(27.0) | 47.0 (29.8) | 48.0(28.0) | 36.0 (19.0) | 40.0 (23.0) |
|  | Mean (SD) | 41.7 (15.5) | 44.0 (14.2) | 45.4 (16.6) | 46.5 (16.1) | 47.8 (16.8) | 47.6 (16.3) | 38.1 (13.3) | 40.7 (14.0) |
|  | Missing (#) | / | / | 8 | / | / | / | / | / |
| Sex | |  | |  | | * | | * | |
|  | Male | 41.0% | 47.9% | 38.2% | 43.9% | 39.1% | 47.3% | 41.5% | 50.2% |
|  | Female | 59.0% | 52.1% | 59.6% | 56.1% | 60.9% | 52.7% | 58.5% | 49.8% |
|  | Missing | / | / | 2.2% | / | / | / | / | / |
| Education | | * | | * | | * | | * | |
|  | High | 43.1% | 41.5% | 56.8% | 57.6% | 55.0% | 58.7% | 58.1% | 52.0% |
|  | Middle | 33.3% | 44.5% | 30.9% | 35.5% | 33.7% | 30.8% | 34.6% | 46.1% |
|  | Low | 22.9% | 14.0% | 12.3% | 6.9% | 11.1% | 10.5% | 6.8% | 1.9% |
|  | Prefer not to answer | 0.4% | / | / | / | 0.2% | / | / | / |
|  | Missing | / | / | / | / | / | / | 0.4% | / |

#### Table 2. Distribution of stringency index by country during data collection dates

|  | Data collection starting date | Stringency index | Data collection end date | Stringency index |
| --- | --- | --- | --- | --- |
| South Africa | 26^th^ May, 2020 | 84.3 | 1^st^ June, 2020 | 84.3 |
| The Netherlands | 22^nd^ April, 2020 | 78.7 | 5^th^ May, 2020 | 78.7 |
| The UK | 22^nd^ April, 2020 | 79.6 | 5^th^ May, 2020 | 79.6 |
| Greece | 22^nd^ April, 2020 | 84.3 | 5^th^ May, 2020 | 84.3 |
| Italy | 22^nd^ April, 2020 | 93.5 | 5^th^ May, 2020 | 93.5 |
| The US | 22^nd^ April, 2020 | 72.7 | 5^th^ May, 2020 | 72.7 |
| Sweden | 26^th^ May, 2020 | 64.8 | 1^st^ June, 2020 | 64.8 |
| Russia | 26^th^ May, 2020 | 78.2 | 1^st^ June, 2020 | 78.2 |

Note to table 2: There were no changes in stringency index in between the dates.

#### Table 3. Multiple linear regression on HRQoL and mental well-being according to non-health and health-related risk factors during the early stage of the COVID-19 pandemic, for healthy (N=10,411) respondents in each country, excluding persons infected with COVID-19

|  |  | **EQ-5D-5L index** | | | | | | | | **EQ VAS** | | | | | | | | **WHO-5 index** | | | | | | | |
| --- | --- | --- | --- | --- | --- | --- | --- | --- | --- | --- | --- | --- | --- | --- | --- | --- | --- | --- | --- | --- | --- | --- | --- | --- | --- |
|  |  | **ZA** | **NL** | **UK** | **GR** | **IT** | **US** | **SE** | **RU** | **ZA** | **NL** | **UK** | **GR** | **IT** | **US** | **SE** | **RU** | **ZA** | **NL** | **UK** | **GR** | **IT** | **US** | **SE** | **RU** |
|  | **N** | 600 | 1477 | 1636 | 552 | 1845 | 1441 | 1176 | 1684 | 600 | 1477 | 1636 | 552 | 1845 | 1441 | 1176 | 1684 | 600 | 1477 | 1636 | 552 | 1845 | 1441 | 1176 | 1684 |
|  | Intercept | 97.2 | 95.6 | 95.2 | 95.7 | 95.8 | 91.6 | 96.8 | 95.9 | 87.5 | 90.0 | 88.7 | 92.7 | 93.0 | 95.6 | 91.2 | 90.6 | 78.7 | 80.2 | 87.0 | 76.5 | 76.3 | 86.7 | 81.9 | 87.3 |
| Age group | |  |  | * |  |  | * | * | * |  |  | * |  | * |  |  | * |  | * |  |  | * | * | * | * |
|  | 25-34 yrs. | -0.5 | 0.5 | 2.7 | 2.0 | 1.6 | 5.1 | -1.2 | 2.1 | 2.6 | 0.7 | 2.3 | 1.8 | -1.1 | -3.2 | -2.7 | 1.5 | -0.6 | 1.5 | 0.4 | 4.2 | -0.1 | 3.5 | 0.2 | 2.2 |
|  | 35-44 yrs. | 0.9 | 0.8 | 3.4 | 1.5 | 0.5 | 4.5 | 0.5 | 0.8 | 2.7 | -0.4 | 4.0 | 2.1 | -1.6 | -3.9 | -0.1 | -0.2 | -2.7 | 3.7 | 0.7 | 4.2 | 2.4 | -0.3 | 3.5 | 4.1 |
|  | 45-54 yrs. | 0.7 | 1.9 | 2.9 | 0.3 | 1.9 | 6.5 | 1.6 | -0.4 | 3.4 | 0.7 | 3.4 | 0.2 | -2.0 | -2.7 | 0.5 | -2.8 | 2.1 | 5.9 | 3.8 | 5.9 | 4.9 | 1.4 | 6.4 | 5.0 |
|  | 55-64 yrs. | 0.3 | 2.6 | 3.1 | 2.5 | 1.6 | 4.4 | 1.5 | -3.8 | 0.2 | 1.6 | 3.5 | -0.1 | -1.7 | -1.9 | 0.8 | -5.2 | -4.5 | 9.3 | 3.2 | 9.6 | 4.7 | 5.2 | 8.8 | 4.5 |
|  | 65-75 yrs. | -2.3 | 1.5 | 0.9 | 3.3 | 0.3 | 4.8 | 5.1 | -2.9 | -4.0 | 4.8 | 2.6 | -4.3 | -6.5 | -1.6 | 3.3 | -4.0 | 5.5 | 11.1 | 6.2 | 2.4 | 6.2 | 4.7 | 11.7 | 8.7 |
| Sex | |  |  |  | * |  |  |  | * | * |  | * |  |  |  |  |  | * | * | * |  | * | * | * | * |
|  | Female | -1.0 | -0.8 | -0.6 | -1.9 | 0.0 | -0.3 | 0.8 | -1.5 | -2.3 | 1.2 | 1.4 | 0.5 | 0.4 | -0.3 | -0.8 | -0.6 | -5.3 | -3.9 | -5.2 | 0.0 | -4.7 | -3.8 | -4.9 | -3.8 |
| Education level | |  |  |  |  |  |  | * |  |  | * |  | * |  |  |  |  |  | * |  |  |  |  | * | * |
|  | Middle | -0.4 | -0.1 | -0.8 | -1.3 | 0.7 | -0.1 | 1.7 | -0.4 | -2.2 | 1.8 | -0.2 | -1.1 | 0.8 | -0.5 | -1.3 | 0.8 | -1.8 | 2.9 | 0.1 | -0.3 | 1.6 | -0.4 | 0.4 | 2.1 |
|  | Low | 3.5 | -0.7 | -1.2 | -1.9 | 1.1 | 0.5 | 2.0 | -3.0 | -13.0 | 1.6 | 3.7 | -7.2 | 1.9 | -0.9 | -2.5 | 0.8 | 6.7 | 3.6 | 5.0 | -10.8 | 1.8 | -1.6 | 1.2 | 3.4 |
| Occupation | |  | * | * | * |  | * | * | * |  |  | * |  |  | * |  | * | * | * | * |  | * | * | * | * |
|  | Student | -2.4 | -3.1 | -2.0 | -3.2 | 0.5 | 3.3 | -2.2 | -0.4 | 1.0 | 0.4 | 0.1 | -1.5 | -0.7 | -4.3 | -3.0 | 0.0 | -6.6 | -6.1 | -7.2 | -6.6 | -3.5 | -3.7 | -3.6 | -6.2 |
|  | Unemployed | -0.3 | -2.9 | -1.9 | -2.0 | -0.8 | -1.3 | -5.1 | -0.5 | -0.6 | -0.3 | -1.4 | -1.1 | -2.1 | 0.0 | -2.6 | -1.5 | -9.9 | -5.3 | -2.5 | -3.0 | -2.2 | -3.0 | -8.3 | -3.9 |
|  | Retired | -2.9 | -1.1 | 1.5 | -1.5 | 0.8 | 0.0 | -4.6 | 2.1 | 5.3 | -0.9 | 1.7 | 2.7 | -0.7 | -0.2 | -1.6 | 3.1 | -4.3 | -0.2 | 3.0 | 2.4 | 2.5 | 1.3 | -1.3 | 2.0 |
|  | Unable to work | 0.3 | -2.5 | -7.6 | -15.7 | -1.3 | -12.9 | -16.8 | -16.8 | -0.5 | -0.8 | -7.7 | -3.6 | -5.8 | -7.7 | -9.6 | -7.6 | -18.6 | 0.4 | -11.1 | -12.7 | -6.9 | -10.5 | -7.5 | -10.0 |
| Income | |  |  |  |  |  |  |  | * | * |  | * |  |  | * |  |  |  |  | * |  | * |  | * | * |
|  | Middle | -0.1 | -0.5 | -0.8 | -1.6 | -0.2 | 0.1 | -2.3 | -1.5 | 1.3 | -2.2 | -1.7 | 1.2 | -0.2 | -1.8 | -1.1 | -1.2 | 2.1 | -1.7 | -5.6 | 3.5 | 0.1 | -2.3 | -0.4 | -3.4 |
|  | Low | -0.4 | -0.6 | -0.4 | -0.1 | -0.4 | -2.7 | -0.9 | -3.3 | 4.0 | -2.0 | -2.8 | 1.4 | 0.3 | -3.3 | -2.2 | -1.8 | 5.0 | -3.4 | -6.4 | 0.1 | -0.7 | 0.3 | 0.4 | -5.0 |
|  | Unwilling to tell | -0.6 | -0.4 | 0.4 | -1.9 | 0.4 | -0.2 | -0.9 | -0.1 | -1.2 | -1.7 | -1.5 | 1.7 | 1.9 | 0.9 | 1.1 | -0.2 | 5.9 | 0.0 | -1.4 | -2.0 | -1.9 | 2.4 | 0.7 | -1.6 |
| Living situation | |  |  |  |  | * |  |  |  |  | * |  |  |  |  |  |  | * |  |  | * |  |  |  |  |
|  | Living alone with children | 0.4 | 0.1 | -0.8 | 3.0 | -3.9 | -1.3 | -0.6 | 1.7 | 5.4 | -4.5 | -1.3 | -0.9 | -0.2 | 0.6 | -0.9 | 1.6 | -1.7 | 0.6 | -1.0 | 2.9 | 1.4 | 3.3 | 3.2 | 1.3 |
|  | Living with other adults | -0.1 | -0.1 | 0.7 | 0.2 | -0.5 | -0.5 | -1.4 | 2.4 | 4.3 | 0.2 | -0.6 | -0.3 | -1.0 | -0.5 | 0.4 | 1.0 | 5.0 | 1.6 | -0.2 | 3.2 | -0.5 | -2.4 | 1.5 | 2.5 |
|  | Living with other adults and children | 0.4 | 0.0 | 0.8 | -0.6 | -0.8 | -0.5 | 0.5 | 2.4 | 3.8 | 0.8 | -0.4 | -1.7 | -1.2 | -0.2 | 1.6 | 1.1 | 6.9 | 1.4 | -0.9 | -1.4 | -0.2 | -1.0 | 3.1 | 3.4 |
|  | Other | 2.0 | -2.0 | -0.7 | -2.3 | -4.5 | -3.2 | -5.7 | 0.8 | 7.0 | -1.8 | -1.0 | -3.3 | 1.1 | -3.1 | 6.7 | 0.1 | 5.9 | 0.7 | -0.6 | -11.0 | -3.9 | -1.9 | -5.4 | 2.1 |
| Smoking | |  |  |  |  | * | * | * | * |  | * |  | * |  |  |  | * | * | * |  | * |  |  | * | * |
|  | Yes | -1.2 | -1.4 | -1.2 | -1.6 | -2.6 | -3.8 | -2.6 | -3.1 | -1.4 | -3.0 | -0.4 | -3.4 | -0.2 | -2.6 | -1.8 | -3.0 | -6.0 | -4.7 | 0.5 | -3.8 | -4.7 | -0.7 | -3.0 | -5.0 |
| Feeling protected against COVID-19 | |  |  | * | * | * | * | * | * | * | * | * | * | * | * | * | * | * | * | * | * | * | * | * | * |
|  | Well | -1.0 | 0.5 | -1.1 | -1.5 | -1.6 | -2.0 | -0.9 | -2.4 | -4.3 | -5.3 | -3.7 | -3.7 | -4.3 | -5.0 | -4.1 | -3.7 | -8.5 | -7.8 | -6.8 | -4.4 | -7.6 | -10.3 | -9.0 | -8.8 |
|  | Reasonably | -2.7 | 0.0 | -3.5 | -5.0 | -2.4 | -0.9 | -2.4 | -4.0 | -7.3 | -6.6 | -7.5 | -6.5 | -6.6 | -7.1 | -6.1 | -8.2 | -18.1 | -12.2 | -16.5 | -12.5 | -13.5 | -14.1 | -14.6 | -15.1 |
|  | Insufficiently | -1.7 | -2.3 | -4.7 | -8.1 | -5.0 | -4.6 | -5.0 | -7.1 | -10.8 | -5.0 | -6.7 | -12.1 | -12.0 | -8.0 | -8.2 | -8.4 | -22.4 | -15.4 | -24.9 | -16.2 | -20.7 | -30.0 | -20.5 | -18.5 |
| Access of healthcare | | * | * | * | * | * | * | * | * | * | * | * | * | * | * | * | * | * | * | * | * | * | * | * | * |
|  | Good/Usually good | -2.6 | -2.1 | -1.4 | -0.1 | -2.2 | -1.1 | -1.6 | -1.9 | -4.0 | -2.5 | -3.4 | -3.0 | -3.6 | -2.9 | -2.2 | -2.7 | -5.7 | -5.0 | -6.1 | -5.6 | -5.9 | -6.8 | -4.8 | -7.8 |
|  | Fair/Sometimes good | -2.2 | -3.3 | -3.2 | -0.5 | -3.9 | -5.6 | -3.6 | -4.3 | -2.4 | -7.0 | -7.7 | -3.4 | -5.3 | -5.9 | -4.5 | -5.2 | -7.5 | -10.1 | -14.0 | -10.4 | -11.1 | -13.1 | -7.3 | -9.4 |
|  | Bad/Usually not good | -0.8 | -8.6 | -3.3 | -5.5 | -5.8 | -25.7 | -3.4 | -6.2 | -4.3 | -7.9 | -5.1 | -4.5 | -8.1 | -13.8 | -1.1 | -5.0 | -11.8 | -17.4 | -17.7 | -8.3 | -19.2 | -8.2 | -7.6 | -12.7 |
|  | Very bad/Never good | 2.6 | 0.6 | -1.0 | -1.4 | -6.3 | -0.1 | -5.1 | -6.6 | 4.5 | -10.3 | -1.1 | -5.1 | -14.7 | -6.2 | -2.0 | -4.6 | -6.8 | -9.7 | -11.7 | -16.9 | -23.1 | -24.0 | -8.1 | -16.1 |

Note to table 3: Reference group: 18-24 yrs., male, high educated, being employed, high household income, living alone, no extra risk of COVID-19 from smoking, feeling very well protected against COVID-19, very good experience with access of healthcare.

#### Table 4. Multiple linear regression on HRQoL and mental well-being according to non-health and health-related risk factors during the early stage of the COVID-19 pandemic, for diseased (N=8.119) respondents in each country, excluding persons infected with COVID-19

|  |  | **EQ-5D-5L index** | | | | | | | | **EQ VAS** | | | | | | | | **WHO-5 index** | | | | | | | |
| --- | --- | --- | --- | --- | --- | --- | --- | --- | --- | --- | --- | --- | --- | --- | --- | --- | --- | --- | --- | --- | --- | --- | --- | --- | --- |
|  |  | **ZA** | **NL** | **UK** | **GR** | **IT** | **US** | **SE** | **RU** | **ZA** | **NL** | **UK** | **GR** | **IT** | **US** | **SE** | **RU** | **ZA** | **NL** | **UK** | **GR** | **IT** | **US** | **SE** | **RU** |
|  | **N** | 394 | 1357 | 1109 | 345 | 1034 | 1263 | 1338 | 1279 | 394 | 1357 | 1109 | 345 | 1034 | 1263 | 1338 | 1279 | 394 | 1357 | 1109 | 345 | 1034 | 1263 | 1338 | 1279 |
|  | Intercept | 98.2 | 86.7 | 97.6 | 88.1 | 90.3 | 90.0 | 85.2 | 84.1 | 88.7 | 84.5 | 87.8 | 84.3 | 80.2 | 90.0 | 78.9 | 79.9 | 87.6 | 75.3 | 81.6 | 62.0 | 70.6 | 83.3 | 73.8 | 66.2 |
| Age group | |  | * |  |  |  | * |  | * |  | * |  |  |  |  | * | * |  | * |  | * |  | * | * | * |
|  | 25-34 yrs. | -2.5 | 6.0 | 0.1 | -2.8 | -1.9 | 1.3 | 4.9 | 0.5 | -2.1 | -1.6 | -3.1 | 2.9 | 1.0 | 0.4 | 5.1 | -1.1 | -1.8 | 2.8 | -2.0 | -3.0 | 3.2 | -1.0 | -0.1 | 3.3 |
|  | 35-44 yrs. | -0.3 | 9.2 | -2.4 | 1.3 | -2.2 | 0.5 | 3.8 | -0.4 | -1.6 | 0.5 | -5.0 | 5.5 | -0.7 | -0.9 | 2.6 | -1.5 | -1.1 | 3.2 | -2.9 | 4.4 | 6.9 | -2.7 | -3.1 | 7.9 |
|  | 45-54 yrs. | -2.6 | 6.7 | -0.1 | 0.0 | 0.4 | 3.4 | 1.9 | 0.4 | -0.2 | -4.0 | -3.0 | 1.8 | -0.8 | -0.2 | 3.3 | -3.5 | 1.9 | 5.6 | -0.4 | 5.8 | 7.5 | -0.5 | 0.8 | 10.3 |
|  | 55-64 yrs. | -4.0 | 9.6 | -1.5 | 3.2 | -2.0 | 5.8 | 3.7 | -1.6 | -1.4 | 1.8 | -3.0 | 5.1 | 0.9 | -1.9 | 3.7 | -4.5 | 6.5 | 9.7 | 3.6 | 16.8 | 9.1 | 2.6 | 5.2 | 11.5 |
|  | 65-75 yrs. | 2.1 | 7.9 | 1.5 | 3.9 | 2.5 | 7.0 | 8.9 | -9.6 | 2.8 | -1.0 | -2.1 | 6.8 | -0.7 | -1.8 | 9.8 | -8.4 | 10.0 | 12.5 | 4.8 | 26.2 | 10.6 | 6.3 | 9.9 | 11.5 |
| Sex | |  | * |  |  |  |  | * | * |  |  |  |  |  |  |  | * | * | * | * |  | * | * | * | * |
|  | Female | 0.4 | -4.0 | 0.3 | -2.5 | -1.6 | -0.6 | -3.6 | 3.6 | 0.4 | -0.4 | -0.5 | 1.0 | 1.2 | 1.4 | -0.4 | 4.7 | -6.1 | -7.1 | -7.1 | -2.6 | -5.5 | -5.7 | -6.3 | -2.6 |
| Education level | |  |  | * |  |  |  |  |  |  |  |  |  |  |  |  |  | * |  |  |  |  |  |  |  |
|  | Middle | 1.7 | -0.3 | -3.7 | 1.1 | -1.2 | -0.3 | -0.6 | -1.9 | 0.8 | 0.0 | -1.8 | -3.8 | -2.0 | -0.4 | -0.7 | -1.3 | 5.3 | 0.8 | -1.3 | -2.7 | 1.2 | 0.6 | 1.2 | 1.1 |
|  | Low | -0.8 | 0.0 | 1.7 | -1.8 | 1.4 | -2.5 | -1.7 | 3.0 | -8.7 | 2.1 | 2.6 | -4.3 | -0.2 | -3.0 | -3.0 | 0.6 | -25.9 | 2.3 | 3.0 | -4.8 | 3.9 | -1.2 | -0.1 | 8.2 |
| Occupation | |  | * | * |  | * | * | * | * |  | * | * | * | * | * | * | * |  | * | * |  | * | * | * | * |
|  | Student | -1.1 | 7.2 | -0.8 | -3.9 | -0.2 | -3.2 | -7.7 | -0.9 | 1.4 | -0.9 | -6.1 | 3.5 | 4.5 | -1.0 | -2.3 | 0.4 | 0.5 | -5.2 | -11.0 | -4.9 | 2.3 | -11.5 | -8.8 | -5.1 |
|  | Unemployed | -1.1 | -2.1 | -4.5 | -3.6 | -3.8 | -3.8 | -5.1 | -3.6 | -1.8 | -1.1 | -0.4 | -2.2 | -5.5 | 0.9 | -3.5 | -1.7 | -8.8 | -3.6 | -5.7 | -5.4 | -7.9 | -7.3 | -4.5 | -4.9 |
|  | Retired | -2.6 | -2.1 | -5.9 | -8.0 | -4.5 | -2.9 | -7.4 | -4.3 | -5.1 | 1.6 | -3.1 | -7.2 | -4.0 | -1.0 | -7.0 | -3.1 | 2.2 | 0.6 | 2.9 | -9.2 | 0.4 | -0.9 | -1.2 | -0.7 |
|  | Unable to work | 0.3 | -16.6 | -29.3 | -6.8 | -25.4 | -19.5 | -25.4 | -31.9 | 1.2 | -9.2 | -14.4 | -12.8 | -19.4 | -10.9 | -16.9 | -10.4 | -2.9 | -8.4 | -13.7 | -1.2 | -10.8 | -10.5 | -9.1 | -15.6 |
| Income | | * |  | * |  |  | * |  |  |  |  | * |  |  | * |  |  |  | * | * |  |  | * |  |  |
|  | Middle | -5.8 | -2.1 | -3.6 | -1.6 | 1.3 | -2.0 | -2.6 | -0.3 | -0.3 | -2.5 | -2.9 | 0.1 | 0.2 | -3.7 | -2.7 | -0.2 | -4.4 | -3.2 | -6.1 | 2.6 | -4.0 | -4.7 | -2.4 | -1.3 |
|  | Low | -3.8 | -2.0 | -6.8 | 1.0 | -0.3 | -7.5 | -1.3 | -1.3 | -6.2 | -1.3 | -7.1 | 2.1 | -0.4 | -7.5 | -3.5 | -1.8 | -6.9 | -3.8 | -11.0 | 1.4 | -4.8 | -3.7 | -2.6 | -4.7 |
|  | Unwilling to tell | -3.9 | -0.7 | -2.4 | 4.6 | 1.2 | -3.1 | -2.4 | 3.2 | 1.4 | -0.2 | -2.0 | 2.4 | -0.3 | -4.1 | -1.9 | -0.1 | -12.4 | -0.8 | -5.3 | 1.0 | -4.0 | -4.6 | -0.6 | -6.2 |
| Living situation | |  |  |  |  |  |  |  |  |  | * |  |  |  |  | * | * | * |  |  |  |  |  |  |  |
|  | Living alone with children | -3.5 | 2.6 | -1.6 | 2.1 | -4.1 | 3.9 | 3.6 | 2.8 | 1.5 | 2.6 | 1.3 | -0.1 | -2.2 | -2.4 | 0.1 | 1.2 | -9.3 | 2.5 | 2.7 | 10.2 | 0.1 | 6.7 | 3.0 | 2.6 |
|  | Living with other adults | -2.6 | 1.5 | -1.2 | -0.3 | -0.1 | -1.5 | 3.9 | 2.3 | 0.7 | 3.3 | 2.2 | -1.9 | -0.7 | -1.1 | 3.8 | 1.2 | -13.2 | 4.0 | 4.2 | 6.6 | -2.3 | 1.3 | 3.7 | 2.1 |
|  | Living with other adults and children | -4.1 | 0.1 | -1.4 | 2.8 | -0.1 | -2.1 | 2.8 | 2.4 | 0.9 | 3.4 | 2.5 | -1.0 | 1.8 | -3.5 | 2.3 | 2.6 | -8.7 | 2.1 | 3.0 | 10.2 | -0.9 | 1.0 | 3.1 | 4.3 |
|  | Other | -3.7 | 2.0 | -3.3 | 6.7 | 0.5 | 0.7 | -3.0 | 1.1 | -0.5 | 6.6 | 7.9 | 5.7 | -3.7 | -0.3 | -4.3 | -0.6 | -12.5 | 2.8 | 8.0 | -2.7 | -5.9 | -1.0 | 2.0 | -0.1 |
| Smoking | |  |  |  |  | * |  |  |  |  |  |  | * |  |  |  |  |  | * | * |  | * |  | * | * |
|  | Yes | 0.7 | -1.6 | -1.5 | 1.1 | -2.8 | -2.6 | -3.6 | -0.6 | -0.2 | -1.2 | -2.1 | -4.3 | -0.8 | -0.1 | -1.0 | 1.1 | -3.5 | -4.1 | -7.4 | -2.0 | -4.8 | -2.7 | -5.0 | -3.2 |
| Chronic conditions | | * | * | * | * | * | * | * | * | * | * | * | * | * | * | * | * | * | * | * | * | * | * | * |  |
|  | 2 | -5.3 | -10.9 | -10.5 | -5.3 | -7.7 | -9.9 | -8.6 | -8.1 | -5.8 | -7.9 | -8.5 | -6.5 | -5.7 | -4.7 | -4.9 | -5.2 | -8.3 | -5.0 | -6.3 | -5.1 | -4.7 | -9.4 | -4.0 | -6.6 |
|  | 3 | -16.2 | -20.1 | -25.2 | -2.9 | -12.1 | -14.0 | -17.5 | -16.5 | -8.5 | -12.2 | -16.1 | -7.6 | -7.8 | -6.2 | -12.9 | -11.9 | -17.2 | -9.3 | -11.5 | -5.9 | -7.7 | -10.7 | -12.1 | -10.1 |
|  | 4 or more | -15.8 | -29.4 | -42.7 | -17.0 | -19.1 | -38.4 | -29.5 | -14.9 | -11.8 | -19.0 | -18.5 | -23.4 | -16.2 | -14.3 | -19.6 | -9.9 | -9.8 | -16.3 | -12.5 | -25.4 | -11.5 | -22.2 | -14.8 | -16.7 |
| Feeling protected | |  |  | * | * | * | * | * | * | * | * | * | * | * | * | * | * | * | * | * | * | * | * | * | * |
|  | Well | -0.6 | -2.5 | -2.7 | 1.1 | 0.7 | -1.6 | 2.8 | 2.5 | -5.1 | -4.6 | -3.6 | -3.3 | 1.7 | -3.6 | -1.8 | -1.0 | -9.1 | -6.6 | -10.1 | -3.8 | -6.2 | -6.7 | -3.5 | -2.3 |
|  | Reasonably | -4.8 | -4.6 | -4.7 | -7.2 | -1.8 | -3.3 | -3.8 | -1.6 | -10.1 | -8.9 | -7.1 | -5.5 | -3.2 | -9.3 | -7.4 | -6.2 | -18.2 | -15.1 | -19.6 | -15.0 | -10.5 | -16.6 | -10.1 | -10.4 |
|  | Insufficiently | -6.4 | -4.3 | -14.3 | -4.4 | -6.9 | -10.0 | -7.2 | -6.3 | -18.1 | -9.8 | -12.6 | -9.3 | -7.0 | -15.0 | -9.8 | -7.9 | -24.3 | -16.9 | -27.1 | -13.6 | -18.4 | -22.7 | -18.5 | -15.6 |
| Access of healthcare | |  | * | * | * | * | * | * | * |  | * | * |  | * | * | * | * | * | * | * | * | * | * | * | * |
|  | Good/Usually good | -2.0 | -1.1 | -2.6 | -3.2 | -3.4 | -2.4 | -1.4 | -2.8 | -1.8 | -2.1 | -2.3 | -0.3 | -2.1 | -2.3 | -2.1 | -1.7 | -3.5 | -4.5 | -4.6 | -4.8 | -6.6 | -8.1 | -6.0 | -3.3 |
|  | Fair/Sometimes good | -3.8 | -9.0 | -5.8 | -8.1 | -7.4 | -6.7 | -2.9 | -3.8 | -3.3 | -6.5 | -4.6 | -4.8 | -5.9 | -8.0 | -3.7 | -2.3 | -15.6 | -14.2 | -8.8 | -9.4 | -12.5 | -13.1 | -9.6 | -4.0 |
|  | Bad/Usually not good | -6.5 | -13.4 | -6.0 | -4.5 | -7.5 | -17.0 | -8.4 | -8.3 | -3.6 | -6.6 | -2.1 | 1.3 | -7.0 | 2.3 | -5.4 | -7.1 | -26.8 | -10.5 | -10.0 | -13.4 | -18.3 | -18.9 | -14.1 | -9.7 |
|  | Very bad/Never good | 1.4 | -10.5 | -9.4 | -11.1 | -5.1 | -52.8 | -14.3 | -12.7 | 3.0 | -3.6 | -6.7 | -1.2 | -4.7 | -29.1 | -11.2 | -5.6 | -17.4 | -14.8 | -9.0 | -20.3 | -15.7 | -19.6 | -15.0 | -16.5 |

Note to table 4: Reference group: 18-24 yrs., male, high educated, being employed, high household income, living alone, no extra risk of COVID-19 from smoking, 1 chronic condition, feeling very well protected against COVID-19, very good experience with access of healthcare.

[1] O. Indicators, "Health at a Glance," 2019.

[2] M. Kaneva, C. J. Gerry, and V. Baidin, "The effect of chronic conditions and multi-morbidity on self-assessed health in Russia," *Scandinavian Journal of Public Health,* vol. 46, no. 8, pp. 886-896, 2018.
